# Variant-aware Cas-OFFinder: web-based *in silico* variant-aware potential off-target site identification for genome editing applications

**DOI:** 10.1093/nar/gkaf389

**Published:** 2025-05-08

**Authors:** Abyot Melkamu Mekonnen, Kang Seong, Hyeran Kim, Jeongbin Park

**Affiliations:** Department of Information Convergence Engineering, Pusan National University, Yangsan, 50612, Republic of Korea; School of Biomedical Convergence Engineering, Pusan National University, Yangsan, 50612, Republic of Korea; Department of Biological Sciences, Kangwon National University, Chuncheon, 24341, Republic of Korea; Department of Information Convergence Engineering, Pusan National University, Yangsan, 50612, Republi c of Korea; School of Biomedical Convergence Engineering, Pusan National University, Yangsan, 50612, Republic of Korea

## Abstract

Genome editing based on CRISPR systems has been widely used in the vast areas of biomedical and agricultural applications. However, identifying the potential off-target sites remains challenging, particularly in individuals with diverse genetic variations. Several *in silico* tools have been developed to predict potential off-target sites, but they have limitations on their performance and scalability. In this paper, we present “Variant-aware Cas-OFFinder,” a novel pipeline based on Cas-OFFinder for identifying potential off-target sites by accounting for individual genetic variants. We benchmarked the pipeline’s improved scalability and performance with the human genome and pepper cultivars, having unique potential off-target sites on each allele at the haplotype level. The web tool is open to all users without a login requirement and is freely available online at https://rgetoolkit.com/var-cas-offinder.

## Graphical Abstract

**Graphical Abstract:**
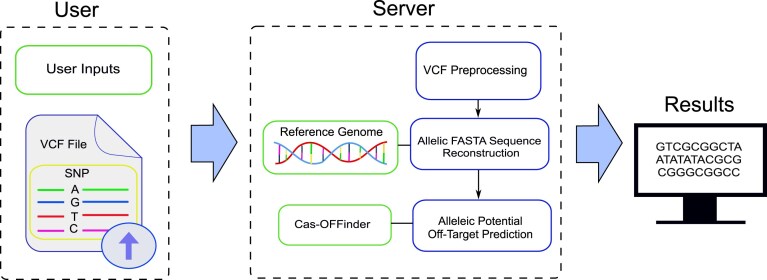

## Introduction

RNA-guided genome editors based on the CRISPR system or beyond CRISPR have strong potential for therapeutics and agricultural fields [1–7]. One of the major challenges in RNA-guided genome editing is its off-target effects [1, 2, 8–11]. In these systems, the guide RNA (gRNA) can direct the effector complex to unintended genomic sites that contain several mismatches, which are referred to as potential off-target sites [3–5, 11].

It is known that off-target effects in RNA-guided genome editing can differ between individuals due to genetic variations such as single-nucleotide polymorphisms (SNPs) and insertions or deletions (indels) [6, 12, 13]. These variations can alter gRNA binding efficiency, potentially introducing new off-target effects or diminishing cleavage efficiency at target sites. Thus, it is crucial to develop personalized off-target site prediction and validation strategies [11, 14].

Several computational tools, such as CRISPRitz, VARSCOT, SNP-CRISPR, and CRISPRme, are designed to identify potential off-target sites considering genetic variants [15–19]; however, CRISPRitz and VARSCOT are unable to perform haplotype-level off-target identification and also lack a web interface. The CRISPRme web tool is restricted to the human genome. Similarly, SNP-CRISPR supports only a limited set of species and is restricted to NGG and NAG Protospacer Adjacent Motif (PAM) types.

In this study, we report a new pipeline based on Cas-OFFinder, a computational tool for identifying potential off-target sites based not only on considering a reference genome but also individual genetic variants. The tool requires a phased, single-sample VCF file as input alongside the reference genome to identify potential off-target sites. Like Cas-OFFinder, our web tool supports rapid identification of potential off-target sites with unlimited mismatches and gRNA length across 557 species and 40 PAM types. PAM types and target genomes are defined via user-editable YAML files, enabling modularity and customizable deployment. To test the applicability of our pipeline, we benchmarked it with human and pepper genomes and found unique potential off-target sites that were never identified by other *in silico* methods.

## Materials and methods

### Reference genome

Reference genomes were primarily obtained from Ensembl (release-112 for vertebrates and release-59 for plants), its affiliates (Bacteria, Fungi, and Protists: release-60), and the NCBI datasets and consistently kept up to date with each new release [20–22].

### Off-target site identification

A VCF file is compressed with *bgzip*, and indexed with *tabix* from samtools (version 1.19.1) [23]. To standardize variant representation, multi-allelic primitives, including gaps and mismatches, are split using the *vcfallelicprimitives* utility available in the vcflib package (version 1.0.3) [24]. This step is done after compressing and indexing the VCF file, ensuring that each variant is correctly resolved.

Next, the VCF records undergo normalization using *bcftools* (version 1.21), which is the standardization of the representation of genetic variants [23]. Multi-allelic sites are further processed using bcftools, which separates alternative alleles into individual VCF records. To reconstruct multi-allelic records while preserving data integrity, the *vcfcreatemulti* utility available in the vcflib package (version 1.0.3) is used [24].

**Figure 1.**
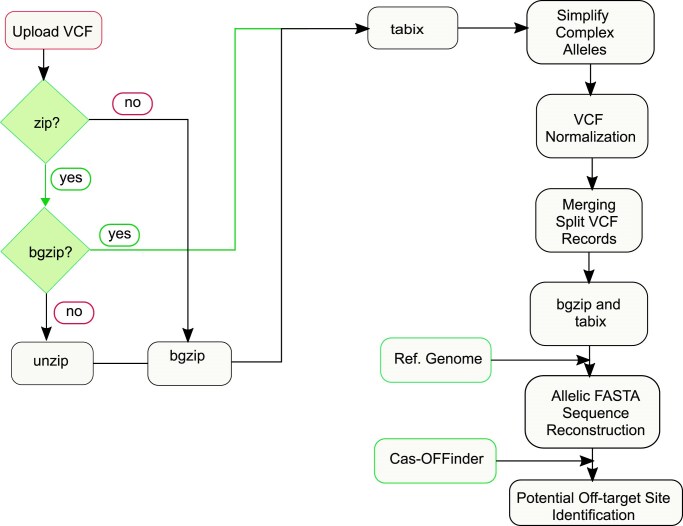
Overview of the Variant-aware Cas-OFFinder algorithm.

The processed VCF file is then used along with the reference genome to reconstruct allelic FASTA files using *vcf2fasta* from the vcflib library (version 1.0.3), enabling sequence-based analyses. Finally, these allelic FASTA sequences are used as input for Cas-OFFinder (version 2.4.1) to identify and quantify potential off-target sites across the entire genome (Fig. 1).

## Results

### Potential off-target site evaluation

To evaluate the potential off-target sites identified with the pipeline, we benchmarked it using pepper and the human genome. For the pepper cultivar, we used our sequencing data to generate a VCF file [25]. For the human genome, we used publicly available variant datasets NA12877 and NA12878 from Illumina’s Platinum Genomes [26].

We analyzed pepper as a test case to validate the pipeline’s performance in a plant genome. To evaluate potential off-target sites, three single guide RNAs (sgRNAs) targeting the CaPAD1 gene were selected from the genomes of pepper cultivars (Table 1), specifically “Dempsey” [25]. Additionally, three sgRNAs targeting the CaPUN1 gene were selected (Table 1) [25]. The Variant-aware Cas-OFFinder results were compared with the original Cas-OFFinder results with up to three mismatches, and unique potential off-target sites were identified.

**Table 1.** Comparison of potential off-target sites identified from the reference genome and allelic FASTA sequence by Cas-OFFinder, CRISPRitz, and Variant-aware Cas-OFFinder in the Dempsey pepper cultivar for NRG PAM using different gRNAs, using a maximum of three mismatches

			Number of potential off-target sites in			
					Variant-aware Cas-OFFinder	
sgRNA sequence	Length	Chromosome	Cas-OFFinder	CRISPRitz	Allele 1	Allele 2
GTGAAATCTAAGTGTAGAGNNN	22	chr1	5	17	17	18
		chr2	3	13	13	12
		chr3	11	21	21	21
		chr4	15	36	35	36
		chr5	6	19	19	18
		chr6	12	27	26	25
		chr7	8	14	14	14
		chr8	3	12	12	12
		chr9	11	26	26	25
		chr10	7	15	15	15
		chr11	4	18	18	18
		chr12	5	13	13	13
TTGTGAAATCTAAGTGTAGNNN	22	chr1	36	622	621	621
		chr2	23	312	313	312
		chr3	22	453	453	453
		chr4	24	427	427	427
		chr5	35	590	590	589
		chr6	25	432	431	432
		chr7	23	590	590	590
		chr8	13	278	279	279
		chr9	24	435	435	435
		chr10	20	461	461	461
		chr11	31	401	398	400
		chr12	28	519	519	519
CTTCACAATTATTCGCCCANNN	22	chr1	2	6	6	6
		chr2	0	4	0	0
		chr3	3	5	5	4
		chr4	0	0	0	0
		chr5	0	0	0	0
		chr6	0	1	0	0
		chr7	1	2	2	2
		chr8	0	1	1	1
		chr9	3	6	6	5
		chr10	2	3	3	3
		chr11	0	2	2	2
		chr12	4	9	9	9
AGATTCAAGAATTGGTACGNNN	22	chr1	4	12	13	11
		chr2	1	5	5	5
		chr3	2	7	7	7
		chr4	4	9	9	9
		chr5	1	13	14	13
		chr6	2	8	8	9
		chr7	6	14	14	14
		chr8	3	4	4	4
		chr9	3	4	4	4
		chr10	6	12	13	13
		chr11	3	13	12	12
		chr12	2	7	7	7
AACCTTCAGTTAGTCGCTANNN	22	chr1	11	16	16	16
		chr2	2	7	7	7
		chr3	10	14	13	13
		chr4	4	6	6	5
		chr5	8	9	9	9
		chr6	7	10	10	10
		chr7	8	13	13	13
		chr8	2	7	7	7
		chr9	12	17	17	17
		chr10	1	3	3	3
		chr11	8	16	15	15
		chr12	5	8	8	8
CACCATAGCGACTAACTGANNN	22	chr1	4	8	6	7
		chr2	0	1	1	1
		chr3	2	3	3	3
		chr4	3	4	4	4
		chr5	0	0	0	0
		chr6	2	4	4	4
		chr7	0	2	0	0
		chr8	0	1	0	0
		chr9	0	2	2	2
		chr10	0	1	1	1
		chr11	2	4	3	4
		chr12	2	3	3	3

We also assessed potential off-target sites within the human genome [26]. For the evaluation, six gRNAs were selected randomly with a mismatch of up to two (Table 2). We identified new potential off-target sites on chromosome 10, which are not found in the reference genome. This evidence suggests that additional genomic locations may be present or absent in the individual genome that will not be recognized in the standard reference sequence.

**Table 2.** Chromosomes with different potential off-target sites between the reference genome and the allelic FASTA file in the human genome (up to two mismatches were allowed in generating data for NA12877.vcf.gz)

		Number of off-target sites in		
			Allelic FASTA files	
sgRNA sequence	Chromosome	Reference	Allele 1	Allele2
GTTTCTCCGACGTGTGCGAGNNN	chr6	1	1	1
CTCCGACGTGTGCGAGAGGANNN	chr6	1	1	1
CTCCATCCTCTCGCACACGTNNN	chr6	1	1	1
CGACGTGTGCGAGAGGATGGNNN	chr6	1	1	1
AACCCAAACGCCCCACCAGGNNN	chr6	1	1	1
CTTTCCGACGCCTCCTGGTGNNN	chr6	1	1	1
CTTTCCGACGCCTCCTGGTGNNN	chr10	0	1	1

### Performance evaluation

To evaluate the runtime performance of our Variant-aware Cas-OFFinder tool, we prepared a single-sample, phased VCF file for the pepper cultivar using the variant caller Octopus. We tested the runtime performance using different gRNA lengths for PAM types NRG and TTTN (Fig. 3).

This performance evaluation was based explicitly on SpCas9 with a 20-nucleotide gRNA sequence for NRG PAM type and AsCpf1 with a 23-nucleotide gRNA sequence for TTTN PAM type, which was selected randomly for the assessment. We used up to two mismatches between the gRNA sequence and the target DNA since many real off-target effects occur with one or two mismatches.

### Comparison with other pipelines

**Figure 2.**
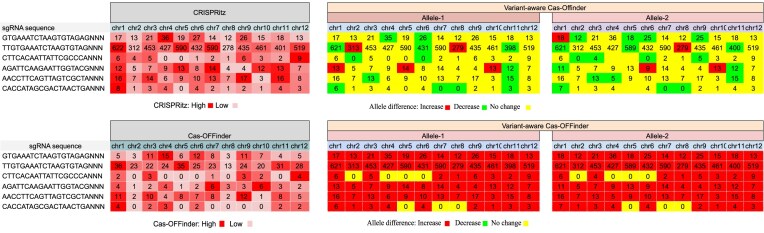
The number of potential off-target sites heatmap in the reference genome and allelic FASTA sequence for different sgRNAs for Variant-aware Cas-OFFinder, Cas-OFFinder, and CRISPRitz.

We compared our pipeline with CRISPRitz, an offline implementation capable of variant-aware off-target identification. We found that our pipeline’s execution time is longer than that of CRISPRitz (Supplementary Table S1). However, CRISPRitz does not support haplotype-aware potential off-target prediction. We compared our results with CRISPRitz and found distinct potential off-target sites (Table 1 and Fig. 2). We observed that CRISPRitz occasionally reports slightly more potential off-target sites than the Variant-aware Cas-OFFinder, due to its inability to account for small indels. Our tool identifies potential off-target sites when alleles of the same chromosome possess both unique and identical SNPs and indels. In addition, the genomic locations of the identified potential off-target sites differ, due to differences in how variant information is incorporated into the reference genome, as our pipeline reports the genomic loci in the reconstructed genome (see Supplementary material).

## Discussion

Using a reference genome alone to identify potential off-target sites has drawbacks because the reference genome may not capture the full genetic diversity within a species. Relying on reference genomes to predict the potential off-target sites may result in missing actual potential off-target sites or incorrectly predicting them, leading to imprecise genome editing.

In this paper, we presented the first web-based, haplotype-aware, variant-aware potential off-target site prediction pipeline, Variant-aware Cas-OFFinder. We showed that it can find unique potential off-targets that are not found by any other available tools. Our pipeline will improve precision in genome editing, having high potential to be applied to various biotechnology fields, such as personalized human therapies and plant trait selection in agricultural applications.

**Figure 3.**
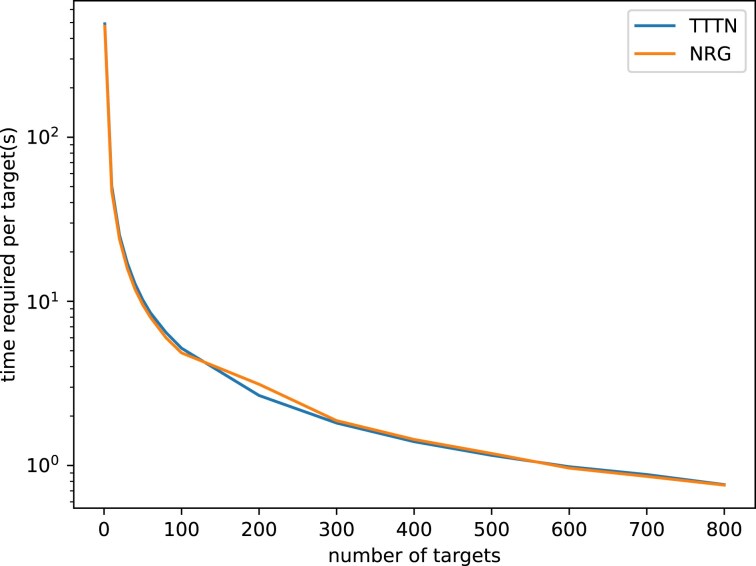
Running time per target site as a function of the number of input target sites on a GPU.

As our pipeline requires allelic FASTA sequences before the potential off-target site prediction, the running time heavily depends on this step. This step is only required once, therefore the average running time per target site decreases as the number of target sites increases (Fig. 3). Although the execution time of our tool is higher than the original Cas-OFFinder due to the time required to construct FASTA sequences from the VCF file and the reference genome, we showed that it still executes in a reasonable time.

The current limitation of the pipeline is that it only considers small variants within the genomic data, including SNPs or small indels, not considering structural variants (SVs). However, we acknowledge that SVs can significantly impact off-target predictions, especially in complex genomes such as those of plants. Still, in many applications, small variants are major concerns, and our pipeline will be useful in these cases.

To facilitate the use of custom genomes, the web interface is fully Dockerized, allowing users to locally deploy the tool with their own genome assemblies. PAM and genome configurations are managed via editable YAML files, supporting customization for species-specific genome editing tasks, including nonmodel organisms and plant cultivars. Additionally, a command-line interface is provided for users who prefer programmatic access and integration into automated workflows. We provide comprehensive documentation on our website for running the pipeline and the web tool locally.

In future work, we aim to extend and validate the pipeline’s compatibility with polyploid genomes (ploidy >2), which are common in many plant species. While the current implementation is designed to be compatible with polyploidy in principle, we are actively testing its performance on diploid genome examples.

## Supplementary Material

gkaf389_Supplemental_Files

## Data Availability

The source code and datasets generated and analyzed during this study are available in our GitHub repository (https://github.com/pnucolab/variant-aware-cas-offinder) and on Zenodo (https://doi.org/10.5281/zenodo.15273194). Additionally, a benchmark code (https://github.com/pnucolab/variant-aware-cas-offinder-benchmark) with all necessary data is available.

## Notes

Present address: Kang Seong, School of Medical Science and Engineering, Korea Advanced Institute of Science and Technology, Daejeon, 34051, Republic of Korea
